# Status of selenium in prostate cancer prevention

**DOI:** 10.1038/sj.bjc.6601974

**Published:** 2004-06-22

**Authors:** G F Combs

**Affiliations:** 1 Center Director, Grand Forks Human Nutrition Research Center, USDA-ARS, Grand Forks, ND 58202-9034, USA

**Keywords:** selenium, carcinogenesis, selenomethionine, methylselenol, prostate, apoptosis, cell cycle

## Abstract

The complete, 13 years, results of the Nutritional Prevention of Cancer Trial have been analysed, causing some speculation over the robustness of the previously reported findings of reduction of cancer risks by supplements of selenium (Se) to a cohort of older Americans. These analyses confirmed that Se supplementation was associated with marked reductions in risks to total (all-site except skin) carcinomas and to cancers of the prostate and colon–rectum. Of those deep-site treatment effects, the most robust was for prostate cancer, which was more frequent, and was confirmed by serum prostate-specific antigen level. Recent subgroup analyses showed Se supplementation reduced risk of cancer mostly among subjects who entered the trial with plasma Se levels in the bottom tertile of the cohort. Other recent findings have demonstrated that Se treatment can promote apoptosis in prostate cancer cells and, possibly, impair their proliferation through antiangiogenic effects. Thus, a body of basic understanding is developing by which one can understand and evaluate the results of the Nutritional Prevention of Cancer and future clinical trials. This understanding also requires inclusion of the mechanisms of Se transport and cellular uptake, so that appropriate inferences can be made from findings from cell culture systems, which tended to use effective Se doses much larger than relevant to cells *in vivo*. Also needed is information on the chemical speciation of Se in foods, so that Se delivery can be achieved in ways that are effective in reducing cancer risk and is also safe, accessible and sustainable.

That selenium (Se), an essential nutrient, may have a role in carcinogenesis was first suggested nearly 40 years ago. That working hypothesis has subsequently developed support from the results of a large number of animal studies that have consistently found Se supplements to be effective in reducing experimental carcinogenesis in virtually every tumour model investigated (see reviews: Combs and Gray, 1998; Combs and Lü, 2001). This body of evidence not withstanding, it was a single report that stimulated current interests in low Se status as a cancer risk factor, and in the potential for Se-containing supplements and foods in cancer prevention. It reported that 10 years of supplementation with a moderate daily dose of the element could substantially reduce cancer risks (Clark *et al*, 1996). Recent analyses of the complete results of what proved to be a 13-year study have raised questions as to the original interpretation of the earlier results and provided the basis for other, larger chemoprevention trials using Se. It is the purpose of this review to discuss the complete trial results in the context of relevant recent work, particularly in research reported in the previous year.

## THE NUTRITIONAL PREVENTION OF CANCER (NPC) TRIAL

The NPC Trial (Clark *et al*, 1996) was a double-blind, randomised, placebo-controlled clinical trial designed to test the hypothesis that a regular oral dose of Se (200 *μ*g day^−1^ as Se-enriched yeast) could reduce the rate of recurrent nonmelanoma skin cancer in a high-risk group of 1312 older Americans living along the eastern seaboard. The results that were first reported (Clark *et al*, 1996) were for the first 10 years of the trial (15 September 1983–31 December 31 1993). They showed no significant effects of Se treatment on the incidences of either basal or squamous cell carcinoma of the skin. The same results, however, showed significant treatment-associated reductions in risks to total cancer incidence (risk ratio (RR)=0.63), total cancer deaths (RR=0.50) and to the incidences of carcinomas at sites other than skin, namely, lung (RR=0.54), prostate (RR=0.37), colon–rectum (RR=0.42) and total nonskin (RR=0.55).

The blinding of the NPC Trial was, in fact, maintained through 31 January 1996 (about the time of publication of the original report (Clark *et al*, 1996)), for a total of more than 13 calendar years. Owing to the untimely death of the project's lead investigator, Dr Larry Clark, analyses of the complete trial results were presented only recently (Duffield-Lillico *et al*, 2002, 2003a, 2003b; Reid *et al*, 2002). With an average of 7.9 years of follow-up per patient, the complete results of the NPC Trial blinded phase provide greater statistical precision than was available for the original analysis (Clark *et al*, 1996) at which time only 6.4 years of follow-up per patient had been achieved.

## RECENT ANALYSES

Analyses of the complete results (Duffield-Lillico *et al*, 2002, 2003a, 2003b; Reid *et al*, 2002) confirmed those conclusions reported earlier in several, but not all, respects. They supported the strongest protective effects of Se detected previously: Se treatment was associated with reduced risks to total cancer incidence (RR=0.63) and to the incidences of carcinomas of the prostate (RR=0.51) and colon–rectum (RR=0.46) (Duffield-Lillico *et al*, 2002). They did not, however, support the earlier finding of a protective effect against lung cancer incidence: risk to cancer at that site was not significantly affected by Se treatment (RR=0.70, *P*=0.18) (Duffield-Lillico *et al*, 2002; Reid *et al*, 2002). Further, analysis of the complete trial data seemed to require the rejection of the previous conclusion that Se treatment did *not* affect the primary end point, which was nonmelanomous skin cancer.

Duffield-Lillico *et al* (2003a), (2003b) reanalysed the original (first 10 year) and complete (13 years) data, in both cases, using a subsample of 1250 patients whose baseline blood samples had been drawn within 4 days of the time each was randomised to treatment. These analyses supported the original finding (Clark *et al*, 1996) that, indeed, Se treatment did not affect the risk of basal cell carcinomas (BCC). In fact, the 13-year data also indicate that Se treatment significantly delayed the diagnosis of the first BCC (Duffield-Lillico *et al*, 2003a, 2003b). However, with the increased statistical power of the complete data set, those analyses also found Se treatment to be significantly associated with *increased* risks to both squamous cell carcinomas (RR=1.31, *P*=0.005) and total nonmelanomous skin cancers (RR=1.22, *P*=0.004).

It is not clear, however, that all of these effects, regardless of their direction, can appropriately be attributed to treatment. This is because it can be expected that at least some cancers, particularly those diagnosed soon after the commencement of the trial, will have resulted from cellular events that occurred prior to the start of the trial, perhaps years earlier. Thus, it is very important to note that analyses of cancer outcomes diagnosed only *after* 2 years of treatment showed no significant treatment effect on SCC incidence (RR=1.21, *P*>0.05). Therefore, it is not clear that Se treatment, which was clearly effective in reducing risks of several deep-site cancers, had any effects on skin cancers.

Vinceti *et al* (2003) commented on the analyses of the complete NPC Trial data by Duffield-Lillico *et al* (2002). As the complete trial was only 25 months longer than the portion originally analysed (Clark *et al*, 1996), Vinceti *et al* (2003) suggested that relatively weaker treatment effects apparent in the former data (Duffield-Lillico *et al*, 2002) must indicate that any cancer protection by Se must occur only in the short term. While their point may obtain to the effects on lung cancer prevalence, they clearly do not with respect to the incidences of total cancers nor of cancers of the prostate and colon–rectum, all of which showed comparable responses to Se treatment in the two periods of follow-up. Neither did Vinceti *et al* (2003) consider the effects of the progressive number of patients lost to follow-up, which, as can be expected, increased as the study continued. Although no participants were lost to vital follow-up (for a total of 9301 person-years), by the end of 13 year blinded period only 36% of patients were still on treatment (Duffield-Lillico *et al*, 2002). Under the ‘intention-to-treat’ paradigm of analysis, this effect could be expected to mitigate detectable treatment effects despite the statistical gains achieved by additional months of follow-up.

Perhaps, the most important finding from the NPC Trial was that the cancer-protective effects of Se treatment were not apparent for all groups of subjects. In the case of total cancer incidence, Se treatment produced significant reductions only in males (RR=0.68, *P*=0.008; *vs* RR=1.14, *P*=0.66 for females) (Duffield-Lillico *et al*, 2002). This finding, however, must be considered in the context that 75% of the trial subjects were men – a fact resulting from the gender-blind recruitment of nonmelanoma skin cancer patients (predominantly male in the US) in seven clinics, the three largest of which were in Veterans Administration Hospitals and had predominantly male patient populations. While that fact may have robbed the study of statistical power relative to outcomes in women, it did the opposite for those of men.

## SELENIUM AND THE PROSTATE

The most robust results of the NPC Trial come from the recent analyses of the complete trial data for prostate cancer incidence, the diagnosis of which was confirmed by analysis of prostate-specific antigen (PSA) in the plasma (Duffield-Lillico *et al*, 2003a, 2003b). These analyses show that, for men with PSA values #4 ng ml^−1^, Se treatment was associated with a 65% reduction in prostate cancer risk (*P*=0.01). In addition, after a finding of elevated PSA, men in the Se-treated group were 40% as likely to undergo biopsy as those in the placebo group (*P*<0.05). Duffield-Lillico *et al* (2003a), (2003b) suggested that this difference may indicate a bias against the detection of prostate cancer in the Se-treated group, but no data were presented to test that speculation. For men entering the trial with PSA >4 ng ml^−1^, there was no significant effect of treatment (RR=0.88, *P*=0.86), nor did Se treatment reduce elevated PSA values or affect the clinical stage or incidence of advanced prostate cancers. These findings would suggest a protective effect of Se treatment against early stage(s) of carcinogenesis; however, there was no indication that Se treatment affected the stage of prostatic disease among men with that diagnosis. In contrast, Rudolph *et al* (2003) found that Barrett's oesophagus subjects with relatively high (upper three quartiles) serum Se levels had reduced risks to developing high-grade dysplasia.

Duffield-Lillico *et al* (2003a), (2003b) also showed that the protective effect of Se treatment against prostate cancer was significant only for subjects who entered the trial with relatively low plasma Se levels. Those entering with plasma Se <106.4 ng ml^−1^ (1.35 nmol l^−1^), that is, in the lowest tertile of that cohort, showed the strongest effect of Se treatment (RR=0.14, *P*=0.002) in reducing the risk of being diagnosed with prostate cancers over the subsequent years of follow-up. Subjects entering in the middle tertile of plasma Se, 106.8–123.2 ng ml^−1^ (1.37–1.58 nmol l^−1^), showed a more modest, but still protective effect of Se treatment (RR=0.39, *P*=0.03); but subjects entering in the highest tertile of plasma Se (>123.2 ng ml^−1^ or >1.58 nmol l^−1^) showed no significant treatment effect (RR=1.20, *P*=0.66). To explore the potential impact of a possible diagnostic bias as indicated above, Duffield-Lillico *et al* (2003a), 2003b) simulated results based on the diagnoses they would have projected had the biopsy rates been comparable between Se and placebo treatment groups; despite a generally attenuated cancer incidence, their analyses showed significant protection by Se in the lowest plasma Se tertile group.

Other recent results support the prostate cancer-protective effect of Se treatment indicated by the NPC Trial, which was designed with nonmelanoma skin cancer as the primary end point. Vogt *et al* (2003) conducted a case–control study of prostate cancer risk in American whites and blacks, finding serum Se concentration to be inversely correlated with prostate cancer incidence. Previously, Brooks *et al* (2001) had shown a similar, inverse association of prediagnostic plasma Se and prostate cancer risk, which was seen only among men with plasma Se levels <118 ng ml^−1^ (<1.51 nmol l^−1^). Van den Brandt *et al* (2003) found that baseline Se status, which they assessed on the basis of toenail Se content, was inversely related to subsequent diagnosis of prostate cancer in a cohort of 1211 men followed for 6.3 years. Like the NPC Trial, van den Brandt *et al* (2003) found the inverse relationship of Se status and prostate cancer risk limited to former smokers, with no significant effects in either ex-smokers or current smokers. Further, the inverse association of Se and prostate cancer risk appeared only among men in the three upper quintiles of baseline toenail Se content. This finding is supported by the previous report (Yoshizawa *et al*, 1998) of a strongly inverse association of toenail Se content and prostate cancer incidence in the Health Professionals Follow-Up Study: men in the highest quintile of toenail Se concentration (determined in 1987) having about half the risk of subsequent prostate cancer (diagnosed in 1989–1994) than those in the lowest quintile.

Inhibition of cell growth has been reported in a variety of cell culture systems, but not until recently have such studies employed prostate cancer cell lines treated with physiologically relevant forms of the element. In studies with androgen-responsive LNCaP cells, the inhibition of cell growth characterised by G(1) phase arrest has been demonstrated in response to treatment with selenomethionine (Venkateswaran *et al*, 2002; Bhamre *et al*, 2003), a predominant form of Se in foods, or methylselenic acid (Jiang *et al*, 2002), a precursor of the putative antitumorigenically active metabolite methylselenol, which formed from the catabolism of selenomethionine and several other food forms of Se. Methylselenic acid has also been shown to cause G(1) arrest in DU145 (Gasparian *et al*, 2002; Wang *et al*, 2002) and JCA1 prostatic carcinoma cells (Gasparian *et al*, 2002). In each case, Se treatment caused caspase-mediated apoptosis, and findings of Se induction of cyclin-dependent kinase inhibitors (Gasparian *et al*, 2002; Venkateswaran *et al*, 2002) and downregulation of PSA transcription (Bhamre *et al*, 2003; Dong *et al*, 2004) suggest antiproliferative effects. Dong *et al* (2002) found selenomethionine treatment to be without effect on androgen-refractory, p53-null, PC3 human prostate cells, which effect could be restored by transfection with the androgen receptor. This would suggest that a functioning androgen receptor may be required for Se sensitivity; however, this is not indicated by the recent results of Zu and Ip (2003) who found methylselenic acid to synergise the growth-inhibitory effect of alpha-tocopherol on PC3 cells. Methylselenic acid was also found by Dong *et al* (2003) to alter the expression by PC3 cells of genes involved in cell cycle progression. Further, selenite, which is reductively metabolised to methylselenides, was effective in inhibiting both primary prostatic tumours and retroperitoneal lymph node metastases in nude mice with established orthotopic PC3 tumors (Corcoran *et al*, 2004). Therefore, the emerging picture is one of induction by Se metabolites of pathways resulting in arrested growth and caspase-mediated programmed death of prostatic cells, reduced androgen signaling and impaired angiogenesis of prostatic tumors.

That Se may have a specific metabolic role in the healthy prostate was suggested several years ago by the identification of a 15 kDa selenoprotein in the rat prostatic glandular epithelium. This protein was shown (Korotkov *et al*, 2001) to form a complex with a larger protein, UDP-glucose:glycoprotein glucosyltransferase that resides in the endoplasmic reticulum and is involved in the control of protein folding. While this finding might suggest a role of Se in the control of protein folding, it would not appear to explain the relatively high amounts of Se in the prostate. Arnold and Thrasher (2003) analysed apparently normal tissue from prostatectomy specimens from six men, finding Se concentrations in the range of 200–267 ng g^−1^ (wet wt.) in five subjects and 421 ng g^−1^ in one subject who reported the regular use of an oral Se supplement. Gianduzzo *et al* (2003) determined Se in specimens obtained from men undergoing transurethral resection for benign prostatic hyperplasia; they found similar prostatic Se concentrations, which were modestly increased by the short-term (1 months) use of an Se supplement (200 *μ*g day^−1^) (Se treated: 241 ng g^−1^ *vs* controls: 196 ng g^−1^; *P*=0.016). Waters *et al* (2003) found similar concentrations of Se in the canine prostate, the levels of which were highly correlated with toenail Se (*P*<0.001). These findings put the prostate in the same category with the kidney as the organs with greatest Se contents.

## IMPORTANCE OF SPECIFIC FORMS OF SELENIUM

The Se supplement used in the NPC Trial, a commercially produced Se-enriched baker's yeast, has not been well characterised. Ip *et al* (2000a), (2000b) reported that the predominant chemical species of Se in a similar product was selenomethionine (SeMet), accounting for more than 80% of total Se, with some 20 other unidentified Se components. Selenomethionine appears to be a dominant species of Se in foods, although there is no evidence that either SeMet or another dominant food species selenocysteine (SeCys), are directly anticarcinogenic. It appears that each must be metabolised to have such effects.

**Figure 1:**
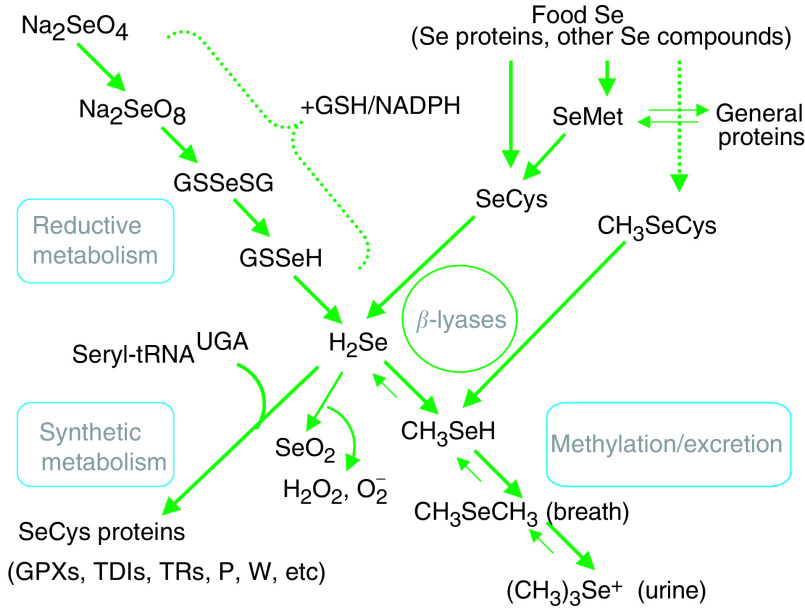
Intermediary metabolism of selenium. (Abbreviations of selenoamino acids: SeMet, selenomethionine; SeCys, selenocysteine; CH_3_SeH: Se-methylselenocysteine. Abbreviations of Se metabolites: GSSeSG, selenodiglutathione; GSSeH, selenoglutathione; H_2_Se, hydrogen selenide; SeO_2_, selenium dioxide; CH_3_SeH, methylselenol; CH_3_SeCH_3_, dimethylselenide; (CH_3_)_3_Se^+^, trimethylselenonium. Abbreviations of specific SeCys-containing proteins: GPXs, glutathione peroxidases; TDIs, iodothyronine 5′-deiodinases; TRs, thioredoxin reductases, P, selenoprotein P; W, selenoprotein W).

Several Se metabolites have been shown to have anti-carcinogenic activities in cell and/or animal model systems: selenodiglutathione (GSSeSG), the reductive metabolite of the oxidised inorganic salts (selenite, selenate), hydrogen selenide (H_2_Se), the common intermediate of that reductive pathway and the catabolism of selenoamino acids, and the methylated selenides ([CH_3_]_*x*_Se), which are excretory forms of the element (see review by Combs and Lü, 2001) (see Figure 1

).

Hydrogen selenide appears to play a central role in Se anticarcinogenesis by way of further metabolism. Its oxidative metabolism produces superoxide anion (O_2_^−^) and hydrogen peroxide (H_2_O_2_), which appear to induce apoptosis (Lü *et al*, 1995). Alternatively, its methylation produces a series of excreted metabolites including methylselenol (CH_3_SeH) which appear to arrest cells in the G_1_ or early S phase and induce apoptosis (Lü *et al*, 1994, 1995, 1996; Zhu *et al*, 1996; Kaeck *et al*, 1997) and to inhibit the cell cycle regulatory enzymes CDK_2_ and protein kinase C (PKC) (Sinha and Medina, 1997; Sinha *et al*, 1999). Ip, Ganther and co-workers (Ip and Ganther, 1990, 1991, 1992a, 1992b; Ip *et al*, 1991, 2000a, 2000b, 2002; Vadhanavikit *et al*, 1993; Dong *et al*, 2002) demonstrated that the CH_3_SeH precursors selenobetaine (CH3SeO2H) and methyl-selenocysteine (CH_3_SeCys) are each more efficacious than selenite in reducing murine mammary tumorigenesis induced by dimethylbenzanthracene. Owing to the di- and trimethylated Se metabolites are very rapidly excreted across the lung (dimethylselenide, [CH_3_]_2_Se) or kidney (trimethylselenonium, [CH_3_]_3_Se^+^), they are not efficacious in such models. Therefore, work has centered on CH_3_SeH precursors (Medina *et al*, 2001; Ip *et al*, 2002). These precursors have been found to inhibit the cell cycle regulatory proteins CDK_2_ and PKC (Sinha and Medina, 1997; Sinha *et al*, 1999) and cyclins D1 and A, while upregulating p27 (Ip and Dong, 2001), to arrest cells in the G_1_ or early S phase, to induce apoptosis (Lü *et al*, 1995, 1996; Kaeck *et al*, 1997; Ip *et al*, 2000a, 2000b), and to inhibit the expression of matrix metalloproteinases in vascular endothelial cells and vascular endothelial growth factor in cancer cells (Jiang *et al*, 1999). From this work it appears that Se *per se* is not anticarcinogenic, but instead that Se supplements and Se-rich foods can have such protective effects to the extent that they contain Se species that are metabolised to yield CH_3_SeH.

## CONCLUSION

How, then, can a normal metabolic role of Se in prostate be rationalised with the clear proapoptotic and antiproliferative efficacy of physiologically relevant Se metabolites? The answer would appear to concern dose. Although the serum/plasma Se concentrations of adequately nourished humans range 70–200 ng ml^−1^ (0.9–2.55 nmol l^−1^), almost all of that Se is bound covalently to proteins which would not be expected to be readily available for uptake by peripheral cells such as those of the prostate. Higher Se doses appear to be needed to produce substantive increases in cellular uptake of Se, particularly of the metabolites thought to be most anticarcinogenically active, the methylselenides that are also rapidly excreted from the body. This hypothesis still needs to be tested in cell culture systems, as it would appear to underlie the general findings that Se is antitumorigenic only at supranutritional doses in animal models, and that Se supplementation can reduce cancer risks in humans consuming nutritionally adequate amounts of the element.

It is, therefore, significant that Duffield-Lillico *et al* (2003a), (2003b) found Se supplementation to be effective in reducing prostate cancer risks in subjects that were nutritionally adequate, but low ranking (lowest one or two tertiles) with respect to Se. It should be noted that the NPC Trial used a form of Se (Se-enriched baker's yeast), the predominant component of which was selenomethionine, a major food form of Se. That this form was effective suggests that the benefits of Se in reducing cancer risk can be achieved at levels of exposure within the normal range, a fact that mollifies the normal safety concerns attending supplementation/fortification efforts. The Duffield-Lillico *et al* (2003a), (2003b) findings also suggest a target blood Se level associated with that protection, of at least 106 ng ml^−1^ (1.35 nmol l^−1^) and, perhaps, 123 ng ml^−1^ (1.58 nmol l^−1^) plasma. Recent analyses of the Third National Health and Nutrition Examination Survey (Niskar *et al*, 2003) indicate that 6 and 52%, respectively, of the US male adult population fall below those levels. As these fractions are probably much higher in most other countries, a large number of people would appear to be able to benefit from moderate increases in Se intake. Delivering Se in accessible and sustainable ways will demand a far better understanding of the chemical species of Se in foods, with particular attention to those species capable of yielding methylselenol metabolically.
